# Case Report: Persistent COVID-19 in a fully vaccinated Japanese man being treated with rituximab and epcoritamab for diffuse large B-cell lymphoma

**DOI:** 10.3389/fmed.2025.1554100

**Published:** 2025-04-30

**Authors:** Masaki Suzuki, Isao Fujioka, Takamitsu Matsushima

**Affiliations:** ^1^Department of Respirology, Kashiwa Kousei General Hospital, Kashiwa, Japan; ^2^Department of Hematology, Kashiwa Kousei General Hospital, Kashiwa, Japan

**Keywords:** persistent COVID-19, diffuse large B-cell lymphoma, rituximab, epcoritamab, SARS-CoV-2

## Abstract

The management of persistent coronavirus disease 2019 (COVID-19) in patients with hematological malignancies who are immunocompromised because of underlying disease or iatrogenic immunosuppression remains clinically challenging. Herein, we report an 84-year-old man with stage 3 diffuse large B-cell lymphoma treated with rituximab and epcoritamab who subsequently developed persistent severe acute respiratory syndrome coronavirus 2 (SARS-CoV-2) infection, despite having received seven doses of COVID-19 mRNA vaccine and remdesivir. The patient was treated with a combination of remdesivir, sotrovimab, and nirmatrelvir/ritonavir, and recovered clinically. SARS-CoV-2 polymerase chain reaction and antigen tests eventually turned negative, and he was discharged after 28 days of hospitalization. This case highlights the challenges associated with managing persistent SARS-CoV-2 infection in immunocompromised patients with hematological malignancies. Combined treatment with antivirals and monoclonal antibodies may be an effective strategy.

## 1 Introduction

Immunocompromised individuals, particularly those with hematological malignancies are susceptible to severe acute respiratory syndrome coronavirus 2 (SARS-CoV-2) infection. Despite the effectiveness of vaccines and antiviral treatment, the risk of coronavirus disease 2019 (COVID-19)-related morbidity and mortality persists, even in the era of the Omicron variant (1). Patients with humoral immunodeficiency, such as those with B-cell malignancies and those receiving anti-CD20 therapy, are particularly susceptible to developing persistent COVID-19. Persistent SARS-CoV-2 infection can lead to decreased viral clearance, prolonged viral shedding, and an increased risk of viral mutations (2–4). The management of persistent COVID-19 in patients who are immunocompromised because of underlying disease or iatrogenic immunosuppression remains clinically challenging. Herein, we report the case of a patient with advanced diffuse large B-cell lymphoma (DLBCL) who developed persistent SARS-CoV-2 infection following treatment with rituximab and epcoritamab.

## 2 Case report

An 84-year-old Japanese man with stage 3 DLBCL received rituximab plus cyclophosphamide, doxorubicin, vincristine, and prednisone therapy, followed by polatuzumab vedotin, bendamustine, and rituximab therapy. Owing to tumor progression, the patient was treated with epcoritamab in June 2024. Although it had been approved for prophylactic use against SARS-CoV-2 infection in Japan, tixagevimab/cilgavimab was not available at the time. The patient had received seven doses of an mRNA-based COVID-19 vaccine according to the schedule recommended in Japan, with the last dose administered in December 2023.

**FIGURE 1:**
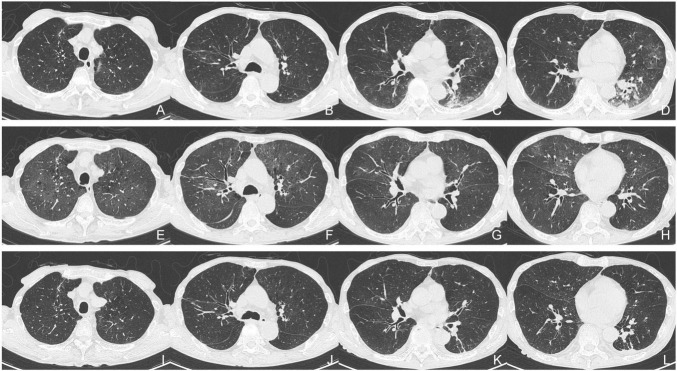
Serial chest computed tomography (CT). **(A–D)** Initial CT scan obtained during the patient’s first episode of COVID-19. **(E–H)** Second CT scan obtained on the patient’s readmission in August 2024. **(I–L)** Follow-up CT scan obtained before the patient’s discharge from hospital.

In July 2024, after the first cycle of epcoritamab, the patient developed a high fever and mild cough. The SARS-CoV-2 rapid antigen test (QuickNavi-COVID19 Ag; Denka Co., Ltd., Tokyo, Japan) was positive, and chest computed tomography (CT) revealed ground-glass opacities in the right lower lobe, left lingual lobe, and left lower lobe of his lungs (Figure 1). The patient was diagnosed with COVID-19 pneumonia and treated with remdesivir for 8 days (200 mg on the first day and 100 mg on the subsequent 7 days), tocilizumab (400 mg on the first day), and cefepime (2 g twice daily for 6 days), before being discharged from hospital on day 9. Nine days after the last administration of epcoritamab in the second cycle (i.e., 40 days after the previous COVID-19 onset), the patient presented with recurrent high fever, cough, and anorexia. Physical examination revealed no evidence of heart failure, including no peripheral edema, weight gain, or wheezing. SARS-CoV-2 antigen and polymerase chain reaction (PCR) tests (Xpert Xpress SARS-CoV-2 Cepheid; Beckman Coulter, Inc., Brea, CA, USA) were positive (cycle threshold [Ct]: 22.8). Chest CT revealed ground-glass opacities in both upper lobes and the right middle lobe of his lungs, although the previously identified abnormal shadows had almost disappeared (Figure 1). The patient was diagnosed with persistent SARS-CoV-2 infection. He had no history of foreign travel, sexually transmitted infections, or inhalational exposures. He was taking fluconazole (100 mg/day) for prophylaxis against fungal infections.

**FIGURE 2:**
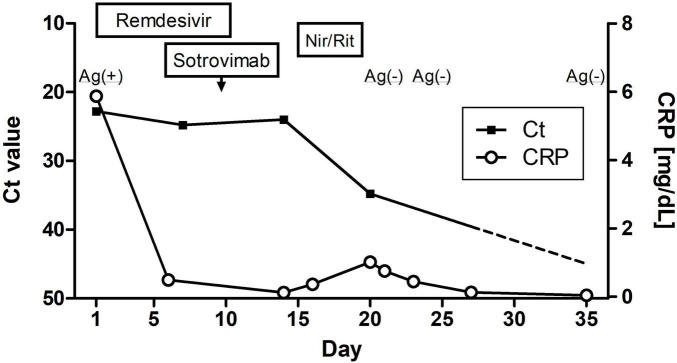
Clinical course of this case showing the changes in SARS-CoV-2 nucleic acid and C-reactive protein levels. Nir/Rit, nirmatrelvir/ritonavir.

The patient’s vital signs on admission revealed fever (body temperature, 38.0°C), tachycardia (125 beats/min), and hypoxia (SpO_2_: 92% breathing ambient air), with a normal blood pressure (127/76 mmHg). His blood test results showed elevated inflammatory marker levels and hypogammaglobulinemia (Table 1). D-dimer levels exhibited a slight elevation, remaining essentially unchanged from the baseline prior to admission. He was treated with intravenous remdesivir for 10 days (200 mg on the first day, followed by 100 mg for 9 days), dexamethasone (6.6 mg/day for 5 days), ceftriaxone (2 g/day for 5 days), and azithromycin (500 mg/day for 3 days) (Figure 2), and oxygen was administered through a nasal cannula. Anticoagulant thromboprophylaxis was not implemented. Tests for autoantibodies associated with connective tissue diseases, serum anti-neutrophil cytoplasmic antibodies, *Mycoplasma* antibody, *Aspergillus* antigen, and cytomegalovirus immunoglobulin M (IgM), and an interferon-γ release assay to screen for tuberculosis were all negative. As the patient had a moderately elevated serum (1→3)-β-D-glucan level, sulfamethoxazole/trimethoprim (3,600/720 mg per day) was administered to treat possible *Pneumocystis jirovecii* pneumonia; however, a sputum PCR test for *P. jirovecii* DNA was negative.

**TABLE 1:** Laboratory test results of the patient on admission.

Parameter	Value	Reference range
Total protein (g/dL)	**5.3**	6.6–8.1
Albumin (g/dL)	**3.2**	4.1–5.1
AST (IU/L)	20	13–30
ALT (IU/L)	10	10–42
ALP (IU/L)	93	38–113
LDH (IU/L)	**449**	124–222
BUN (mg/dL)	15.0	8.0–20.0
Creatinine (mg/dL)	0.80	0.65–1.07
Sodium (mEq/L)	**130**	138–145
Chloride (mEq/L)	**97**	101–108
Potassium (mEq/L)	3.8	3.6–4.8
Calcium (mmol/L)	**8.7**	8.8–10.1
CRP (mg/dL)	**5.87**	0.00–0.14
PCT (ng/mL)	**0.20**	< 0.05
IgG (mg/dL)	**483**	861–1,747
IgA (mg/dL)	**56**	93–393
IgM (mg/dL)	**10**	33–183
sIL2-R (U/mL)	**2,256**	145–519
Blood glucose (mg/dL)	**137**	73–109
HbA1c (%)	5.5	4.9–6.0
BNP (pg/mL)	**33.3**	< 18.5
*Mycoplasma* Ab	Negative	
(1→3)-β-D-glucan (pg/mL)	**123.6**	< 11.0
*Aspergillus* Ag	< 0.2	< 0.2
Sputum PCR for *P. jirovecii* DNA	Negative	
Cytomegalovirus IgG (AU/mL)	**104**	< 6.0
Cytomegalovirus IgM (index)	0.13	0.00–0.84
T-SPOT.TB	Negative	
HIV Ag/Ab	Negative	
KL-6 (U/mL)	**860**	< 500
SP-D (ng/mL)	**254**	< 110
White blood cell count (cells/μL)	5,400	3,300–8,600
Neutrophils (%)	73	42–74
Lymphocytes (%)	**17**	20–60
Monocytes (%)	9	0–12
Red blood cell count (× 10^4^/μL)	**359**	435–555
Hb (g/dL)	**10.5**	13.7–16.8
Hct (%)	**31.2**	40.7–50.1
MCV (fL)	86.9	83.6–98.2
MCHC (g/dL)	33.7	31.7–35.3
Platelet (× 10^3^/μL)	174	158–348
PT-INR	1.01	
aPTT (s)	30.3	24.0–34.0
Fibrinogen (mg/dL)	**471**	150–400
FDP (μg/mL)	3.0	< 5.0
D-dimer (μg/mL)	**1.4**	< 1.0

Bold indicates values outside the normal range. Ab, antibody; Ag, antigen; ALP, alkaline phosphatase; ALT, alanine transaminase; aPTT, activated partial thromboplastin time; AST, aspartate transaminase; BNP, brain natriuretic peptide; BUN, blood urea nitrogen; CRP, C-reactive protein; FDP, fibrin/fibrinogen degradation products; Hb, hemoglobin; HbA1c, glycated hemoglobin; Hct, hematocrit; HIV, human immunodeficiency virus; Ig, immunoglobulin; KL-6, Krebs von den Lungen-6; LDH, lactate dehydrogenase; MCV, mean corpuscular volume; MCHC, mean corpuscular hemoglobin concentration; *P. jirovecii*, *Pneumocystis jirovecii*; PCT, procalcitonin; PT-INR, prothrombin time international normalized ratio; sIL2-R, soluble interleukin-2 receptor; SP-D, surfactant protein D.

Although the patient’s high fever and hypoxia resolved, a follow-up SARS-CoV-2 PCR test remained positive (Ct: 24.8) (Figure 2), and his symptoms of cough, fatigue, and anorexia persisted. On day 9 of hospitalization, he received sotrovimab (500 mg) monoclonal antibody treatment. On day 14, another SARS-CoV-2 PCR test was again positive (Ct: 24.0). Nirmatrelvir/ritonavir was administered on day 15. On day 21, the SARS-CoV-2 antigen and PCR test results were negative (Ct: 34.8). By day 24, the CT abnormalities had almost disappeared (Figure 1), and the patient was discharged on day 28 of hospitalization. One month after discharge, a follow-up PCR test for SARS-CoV-2 remained negative (Ct > 45.0) and there was no evidence of recurrent abnormalities on the CT scan. The CT severity of lung abnormalities was evaluated by three clinicians using a semi-quantitative scoring method as described by Pan et al. (5). The mean severity scores were 10.7, 11.0, 7.3, and 4.0 in the initial CT, second CT (admission in this report), pre-discharge follow-up, and 1-month post-discharge follow-up, respectively. The scores for each lung lobe are shown in Supplementary Table 1.

## 3 Discussion

Anti-CD20 therapy is frequently used to treat B-cell hematological malignancies. However, anti-CD20 agents can lead to prolonged immunosuppression that makes patients more susceptible to infection, including SARS-CoV-2 infection (2, 3). Rituximab, a commonly used immunosuppressive drug targeting B-cells, is associated with reduced response to SARS-CoV-2 vaccination (6). Patients with hematological malignancies who receive anti-CD20 therapy may have a reduced immune response to SARS-CoV-2 infection for as long as 12 months, which increases their risk of persistent COVID-19 (7). Epcoritamab is a novel bispecific antibody that targets both CD20 and CD3 and activates T-cells to target and eliminate CD20-expressing cells (8). The effect of epcoritamab-induced CD20 depletion in SARS-CoV-2 infection remains unclear, and there are only a few reports on the clinical course of persistent SARS-CoV-2 infection following rituximab and epcoritamab treatment. Faxén and Edvinsson (9) described a case of persistent COVID-19 treated using a combination therapy of remdesivir, tixagevimab/cilgavimab, and nirmatrelvir/ritonavir. Longo et al. (10) reported three cases of persistent COVID-19 and highlighted the effectiveness of combination therapy with antiviral agents and monoclonal antibodies. In our case, despite receiving rituximab and epcoritamab, which may have exerted an additive immunosuppressive effect, the patient recovered after sequential administration of remdesivir, sotrovimab, and nirmatrelvir/ritonavir.

Identifying patients who are most likely to experience persistent COVID-19 and establishing appropriate diagnostic criteria and management strategies for patients at high risk remain major clinical challenges. As humoral immunity plays a crucial role in the clearance of SARS-CoV-2, patients with B-cell depletion caused by conditions such as hematological malignancy, anti-CD20 therapy, hematopoietic stem cell/solid organ transplant, and common variable immunodeficiency, are more susceptible to persistent SARS-CoV-2 infection (1, 2). Although local protocols for the management of prolonged COVID-19 in patients with severe immunosuppression have been introduced (11), the optimal diagnostic evaluation and therapeutic strategies for patients with different degrees of immunodeficiency remain unclear. Moreover, although the US National Institutes of Health COVID-19 treatment guidelines acknowledge the occurrence of prolonged viral shedding in immunocompromised individuals (12), standardized clinical terminology for this phenomenon has not yet been established. Given our patient’s clinical course, prolonged viral shedding, symptoms, and CT findings, we diagnosed him with persistent COVID-19 based on the proposed criteria for persistent COVID-19 (11, 13).

Consensus regarding the optimal treatment strategy for persistent SARS-CoV-2 infection in patients who are immunocompromised, such as those undergoing B-cell depletion therapy, is lacking. Remdesivir suppresses viral replication by blocking the RNA-dependent RNA polymerase, which is essential for SARS-CoV-2 replication (14). Similarly, nirmatrelvir inhibits 3CL protease, an enzyme necessary for SARS-CoV-2 viral replication, and ritonavir inhibits its degradation. Dexamethasone, in combination with remdesivir is effective for treating COVID-19 (15). However, the potential for adverse outcomes due to steroid-induced suppression of interferon production and prolonged viral shedding has concerns (16). Therefore, cautious use of dexamethasone in combination with remdesivir with attention to dosage and duration is warranted in patients with hematological malignancies. The use of single antiviral agents for persistent SARS-CoV-2 infection is associated with a risk of drug resistance and promotes viral evolution (4). Prolonged use of nirmatrelvir/ritonavir treatment has been reported to be effective for treating persistent SARS-CoV-2 infection (17). However, clinically significant resistance to remdesivir and nirmatrelvir/ritonavir has been reported, particularly in patients with hematological malignancies and persistent SARS-CoV-2 infection (14). Recent reviews and case series have highlighted the effectiveness of therapies involving a combination of antiviral agents and monoclonal antibodies in patients who are immunocompromised with persistent COVID-19 (9, 10, 18–20). Both sequential and simultaneous combination therapies have been reported to be safe and effective (10). The Japanese guidelines for COVID-19 do not mention simultaneous combination therapy with antiviral agents (21). In this case, we used sequential combination therapy, which resulted in a favorable outcome. Intravenous immunoglobulin (IVIg) boosts and modulates the immune system and helps to fight infection in patients with immunodeficiency. Although the effectiveness of IVIg against persistent COVID-19 has not been confirmed, Maruki et al. (22) reported successful use of IVIg and antiviral agents to treat persistent COVID-19 in an immunocompromised patient who was receiving CD20-depleting therapy for follicular lymphoma. Convalescent plasma may offer therapeutic benefits for persistent COVID-19 (18), but its availability in general medical practice is limited. Optimizing personalized patient care and the choice and prioritization of drug combinations is challenging owing to the heterogeneity of immunosuppression according to the type and severity of the underlying disease. Further research in this area is warranted.

Previous studies have demonstrated the effectiveness of neutralizing antibodies for treating persistent COVID-19 (9, 10, 18–20). Although sotrovimab has been reported to be less effective at neutralizing SARS-CoV-2 variants BA.2.12.1, BA.4, and BA.5 *in vitro* (23), in real-world settings, it continued to be effective at preventing severe disease and complications during the period when the BA.2 and BA.5 variants were predominant (24). In patients with hematological malignancy, sotrovimab substantially boosts neutralizing antibody titers, even in those with inadequate humoral immune responses to COVID-19 vaccination (25). These studies suggest that sotrovimab may be a valuable therapeutic option against SARS-CoV-2 Omicron subvariants. In our patient, sotrovimab was administered sequentially owing to the unavailability of casirivimab/imdevimab.

Persistent COVID-19 can present with or without abnormal pulmonary findings, and the severity of respiratory decompensation varies widely (9, 10, 19). Frequently, multiple ground-glass opacities are observed on CT scans, and migration of airspace opacities, as demonstrated in this case, may also be observed (26) (Supplementary Table 1). In cases of ground-glass opacities that appear after the onset of COVID-19, the differential diagnosis includes persistent SARS-CoV-2 infection, as well as infections induced by a broad spectrum of pathogens, non-infectious diseases, pulmonary edema, and exacerbation complicating pre-existing interstitial lung disease. The wide range of conditions to consider in the differential diagnosis contributes to the diagnostic challenge. Challenges to diagnosing persistent COVID-19 include the positioning of imaging patterns and predicting cases of severe or refractory disease that require close monitoring. Even cases with a relatively mild but persistent clinical course of COVID-19 pneumonia can be fatal (10, 27), highlighting the need for further research in this field.

This study has some limitations. First, genetic sequencing of the SARS-CoV-2 virus was not performed in this case. According to Japanese nationwide surveillance data, the Omicron variant JN.1 and its subvariants accounted for approximately 95% of SARS-CoV-2 strains circulating in Japan in July 2024 (28), consistent with global trends (29). Second, no lower respiratory tract specimens (e.g., bronchoalveolar lavage fluid) were collected from the patient; therefore, the possible involvement of other respiratory pathogens cannot be ruled out in this case. However, except for elevated (1→3)-β-D-glucan levels, no other laboratory findings suggested the presence of other infections. Decisions regarding further testing should be personalized based on each patient’s unique immune status and the invasiveness of the proposed diagnostic procedures.

This case highlights key aspects of persistent COVID-19 in immunocompromised patients. Although patient backgrounds and treatment strategies vary, most cases are mild, with favorable outcomes from antiviral monotherapy or combination therapy. Notably, viral load appears to rapidly decrease following clinical improvement (10). Few reports are available of persistent infection following rituximab and epcoritamab treatment (9, 10, 30, 31). Among them, Bay et al. (30) suggested that intermittent remdesivir monotherapy may induce resistance through ORF1b:C455F mutation. As with our patient, most patients improve with prolonged (sequential or simultaneous) nirmatrelvir/ritonavir-based therapy. Although Breeden et al. (31) reported pneumonia associated with persistent SARS-CoV-2 infection, to our knowledge, our report is the first report migratory ground-glass opacities observed on serial high-resolution CT scans. Persistent COVID-19 in immunocompromised patients is diverse and clinically challenging to manage. More clinical research is needed for optimal practice.

In conclusion, in immunocompromised patients, particularly those with B-cell hematological disorders who are undergoing chemotherapy, are at risk of persistent SARS-CoV-2 infection. Although no standardized guidelines are available for treatment of persistent SARS-CoV-2 infection, combination therapy should be considered in immunocompromised patients to enhance the effectiveness of treatment and reduce the risk of prolonged viral shedding and the emergence of new SARS-CoV-2 mutations.

## Data Availability

The raw data supporting the conclusions of this article will be made available by the authors, without undue reservation.
