# Supplementary material for: Case Report: Persistent COVID-19 in a fully vaccinated Japanese man being treated with rituximab and epcoritamab for diffuse large B-cell lymphoma
Source: Front Med (Lausanne). 2025 Apr 30;12:1554100. doi: 10.3389/fmed.2025.1554100 (PMC12075373; doi:10.3389/fmed.2025.1554100)
Supplement: Supplementary file 1 [file Table_1.docx]

**Supplementary Table 1.**

CT severity scores for the total lung and each lobe at each time point.

|  | CT severity score, mean (range)^a^ | | | |
| --- | --- | --- | --- | --- |
|  | Initial CT^b^ | Second CT^c^ | Follow-up CT before discharge | Follow-up CT after discharge^d^ |
| Right upper lobe | 1.3 (1–2) | 3.0 (2–5) | 2.0 (2–2) | 1.3 (1–2) |
| Right middle lobe | 1.0 (1–1) | 3.0 (2–5) | 1.7 (1–3) | 1.3 (1–2) |
| Right lower lobe | 1.7 (1–2) | 0.7 (0–1) | 1.0 (1–1) | 0.7 (0–1) |
| Left upper lobe | 2.3 (2–3) | 3.0 (2–5) | 1.3 (1–2) | 0.3 (0–1) |
| Left lower lobe | 4.3 (4–5) | 1.3 (1–2) | 1.3 (1–2) | 0.3 (0–1) |
| Total lung | 10.7 (10–11) | 11.0 (7–13) | 7.3 (6–10) | 4.0 (4–4) |

^a^ The mean and range are the mean and range of the scores assigned by three clinicians using the same set of CT images.

^b^ The initial CT scan was taken during the patient’s first COVID-19 episode in July 2024.

^c^ The second CT scan was taken on the readmission to hospital in August 2024.

^d^ The follow-up CT scan was taken 1 month after discharge.

**Figure Legend:**

The scores are presented as the mean (range). The severity scores for each of the five lung lobes were calculated based on the extent of anatomical involvement (ground-glass opacity, crazy paving, and consolidation), using the following scale: 0, no involvement; 1, <5% involvement; 2, 5–25% involvement; 3, 26–50% involvement; 4, 51–75% involvement; and 5, >75% involvement. Image analysis was performed independently by three clinicians, with 13, 30, and 46 years of experience, respectively, including two radiologists, at four time points: the initial CT scan during the patient’s first COVID-19 episode in July 2024, the second CT scan on the patient’s readmission in August 2024, the pre-discharge follow-up CT scan, and the follow-up CT scan 1 month after discharge.
